# Unguis incarnatus – konservative oder operative Therapie? Ein praktischer Behandlungsalgorithmus

**DOI:** 10.1007/s00113-020-00903-6

**Published:** 2020-10-27

**Authors:** N. Moellhoff, H. Polzer, S. F. Baumbach, K. G. Kanz, W. Böcker, V. Bogner-Flatz

**Affiliations:** 1 Abteilung für Hand‑, Plastische und Ästhetische Chirurgie, Klinikum der Universität München, LMU München, Pettenkoferstr. 8a, 80336 München, Deutschland; 2 Klinik für Allgemeine, Unfall- und Wiederherstellungschirurgie, Klinikum der Universität München, LMU München, München, Deutschland; 3 Klinik und Poliklinik für Unfallchirurgie, Klinikum rechts der Isar, Technische Universität München, München, Deutschland

**Keywords:** Eingewachsener Zehennagel, Onychocryptosis, Emmert-Plastik, Podologie, Phenolkauterisierung, Ingrown toenail, Onychocryptosis, Emmert-plasty, Podiatry, Phenol cauterization

## Abstract

Der *Unguis incarnatus* ist ein häufiges Krankheitsbild, mit dem sich Patienten in der Hausarztpraxis, der dermatologischen Klinik oder der chirurgischen Notaufnahme vorstellen. Häufig führt die inkonsequente konservative Therapie oder die falsch-indizierte operative Intervention zu langwierigen und komplikationsreichen Verläufen, inklusive Rezidiven. Die Patienten sollten über die Komplexität des Nagelorgans aufgeklärt werden, um der Banalisierung der Erkrankung vorzubeugen, und eine entsprechende Compliance in der Therapie zu erreichen. In diesem Manuskript wird die sachgerechte Versorgung des *Unguis incarnatus* im Sinne eines praktischen Behandlungsalgorithmus dargestellt. Die konsequente konservative Therapie ist bei akutem *Unguis incarnatus* mit milder Ausprägung die Therapie der ersten Wahl mit guten Behandlungsergebnissen. Nagelerhaltende operative Eingriffe kommen bei moderaten/schweren akuten Formen zum Einsatz. Der chronische *Unguis incarnatus*, ohne floride Infektion, stellt eine elektive Operationsindikation dar. Sowohl bei den nagelerhaltenden Eingriffen als auch bei erweiterten operativen Maßnahmen ist eine chirurgische Operationsaufklärung obligat.

## Abstract (en)

Unguis incarnatus, an ingrown toenail, is a common condition in primary care, which is encountered by various medical professions. Inconsistent conservative treatment and nonindicated surgical treatment often result in complications and recurrence of the disease. Patients must be thoroughly informed about the complexity of the nail organ. This is a prerequisite to prevent trivialization of the disease and to achieve appropriate patient compliance for treatment. In this article a practical diagnostic and treatment algorithm for unguis incarnatus is presented*. *In mild cases of acute unguis incarnatus a consistent conservative treatment is the first-line strategy showing promising results. In cases of moderate to severe forms of acute unguis incarnatus, surgical procedures that preserve the nail matrix should be applied. For cases of chronic unguis incarnatus without an acute infection, elective partial matrixectomy can be indicated. Prior to any surgical intervention, detailed informed consent must be obtained from the patients.

## Hinführung zum Thema

Die Behandlung eingewachsener Zehennägel – was zunächst nach Banalität klingt, stellt Ärzte in der klinischen Praxis häufig vor eine Herausforderung. Die Erwartungshaltung des Patienten gegenüber dem Arzt ist oft hoch, es handelt sich schließlich „nur“ um einen eingewachsenen Zehennagel. Von ärztlicher Seite ist hier jedoch äußerste Vorsicht geboten, nachdem konservative und operative Maßnahmen nicht selten von Misserfolg geprägt sind, insbesondere, wenn diese aufgrund der vermeintlichen Einfachheit leichtfertig von unerfahrenen Behandlern durchgeführt werden [1].

## Hintergrund

**Figure 1:**
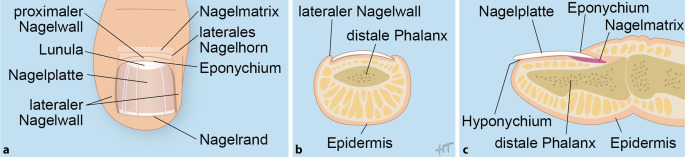

**Figure 2:**
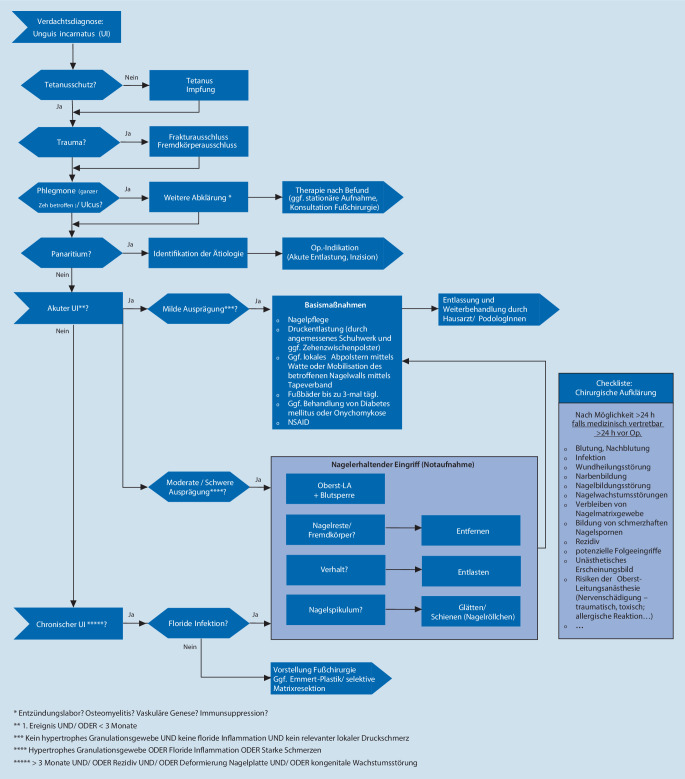

Der Nagelapparat ist anatomisch komplex und besteht aus den im Folgenden aufgeführten Strukturen (Abb. 1; [2]). Die Nagelmatrix liegt unter dem proximalen Nagelwall und produziert die Nagelplatte. Sie ist im proximalen Bereich des Nagels als sog. Lunula sichtbar durchschimmernd. Die lateralen Nagelplattenhörner liegen unterhalb des lateralen, proximalen Nagelwalls. Das Eponychium ist die Unterseite des proximalen Nagelwalls und haftet fest am darunter liegenden Nagel. Die lateralen Nagelwälle schließen den Nagel seitlich ein. Das Nagelbett dient als Gleit- und Haftlager für den Nagel. Es wird distal vom Nagelisthmus begrenzt, der in das Hyponychium unter dem freien Nagelrand übergeht [3].

Der *Unguis incarnatus* ist eine der häufigsten Erkrankung des Nagelorgans [4, 5]. Die Prävalenz beträgt zwischen 2,5 und 5 % [6, 7], mit einer Häufung im Jugend- und im jungen Erwachsenenalter. Männer sind dabei etwa doppelt so häufig wie Frauen betroffen.

Ein eingewachsener Zehennagel entsteht, wenn die Nagelplatte die periunguale Haut irritiert. Dies führt im Sinne einer Fremdkörperreaktion zu Inflammation und Hypergranulation mit sekundärer Infektion des betroffenen Nagelwalls. Klinisch präsentiert sich der Patient mit Schmerzen, Rötung und Schwellung des Nagelwalls oder des gesamten Zehs. Eine Abszedierung kann vorliegen. Meist ist die lateralseitige Großzehe betroffen [7–9].

Die häufigsten Ursachen für den *Unguis incarnatus*, v. a. bei jungen Patienten, sind das falsche Schneiden der Nägel und das Tragen von zu engem Schuhwerk. Durch falsches rundes Zurückschneiden des Nagels kann im Bereich des seitlichen Nagelwalls eine spitze Nagelecke – auch Spikulum genannt – entstehen, die folglich in den Nagelwall einwächst. Enges Schuhwerk führt zu einem vermehrten interdigitalen Druck zwischen Hallux und zweiter Zehe. Dadurch wird bei der Großzehe die laterale Nagelplatte gegen den lateralen Nagelwall gedrückt, was wiederrum zu einer lokalen Irritation führen kann. Weitere Ursachen sind mangelnde Nagelpflege/Hygiene, wiederholte Traumata am Nagelorgan, Hyperhidrose, Nagelpilzbefall, Diabetes mellitus, Immunsuppression, Schwangerschaft, die Einnahme von EGF-Rezeptor-Inhibitoren oder mechanische Ursachen durch Fußfehlstellungen [7, 10–13]. Auch Hyperostosen bzw. Osteophyten können durch Druck auf die Nagelmatrix oder das Nagelbett zu Nagelfehlbildungen („pincer nail“) und sekundären Irritationen führen [14]. Des Weiteren ist eine Reihe endogener Ursachen beschrieben: Nageldeformitäten wie die *Onychogrypose* [15] und anlagebedingte anatomische Varianten können einen *Unguis incarnatus* begünstigen. Eine positive Familienanamnese ist dann in den meisten Fällen zu beobachten [5, 16].

Der *Unguis incarnatus *ist ein häufiges Krankheitsbild, mit welchem verschiedenste medizinische Fachrichtungen konfrontiert sind. Trotz der erstaunlichen Menge an Literatur fehlt ein differenzierter Diagnose- und Behandlungsalgorithmus [17, 18]. Im Rahmen dieser Arbeit soll die vorhandene Evidenz dargestellt und das in der chirurgischen Notaufnahme praktisch angewandte Vorgehen präsentiert werden (Abb. 2).

## Diagnostik

Zunächst sollten im Rahmen der initialen Vorstellung, neben der Anamnese, der Tetanusschutz überprüft sowie eine mögliche traumatische Ursache ausgeschlossen werden. Sollte sich in der anschließenden klinischen Untersuchung eine Phlegmone der gesamten Zehe, ein Ulkus oder ein Panaritium zeigen, müssen die Ätiologie identifiziert und entsprechend weitere diagnostische und therapeutische Maßnahmen initiiert werden.

Liegt ein *Unguis incarnatus* vor, empfehlen wir die Unterscheidung in akute und chronische Formen. In der Literatur wird eine Vielzahl von Klassifikationen postuliert. Die Autoren orientieren sich an den Stadieneinteilungen nach Mozena et al. und Martínez-Nova et al. ([19, 20]; Tab. 1). Diese basieren auf dem Grad der Inflammation, der Hypertrophie des lateralen Nagelwalls und der Deformität der Nagelplatte.

**Table 1:** 

Stadieneinteilung	Klinisches Bild
I	Erythem, Ödem, Schmerz bei Druck auf lateralen Nagelwall, keine Wucherung des Nagelwalls über die Nagelplatte
IIa	Symptome wie in I, vermehrte Schmerzen, Ödem, Erythem, Hyperästhesie, seröses Wundexsudat, ggf. Infektion, Wucherung des Nagelwalls über die Nagelplatte <3 mm
IIb	Symptome wie in IIa, ggf. Infektion und Abszedierung mit Wucherung des Nagelwalls über die Nagelplatte >3 mm
III	Chronische Hypertrophie und Hypergranulation, Gewebe bedeckt einen Großteil der Nagelplatte, ggf. Abszess, Ulzeration, Deformierung
IV	Chronische Deformierung der Nagelplatte, beider lateraler Nagelwälle, Hypertrophie mit Bildung eines distalen Nagelwalls

Als akuten *Unguis incarnatus *definieren wir das erstmalige Auftreten mit einer Symptomdauer von weniger als 3 Monaten. Dies entspricht den oben genannten Stadien I und II [19, 20]. Entscheidend für die Therapie ist die weitere Subklassifizierung in milde und moderate/schwere Ausprägungsformen (Abb. 2).

Bei der chronischen Form handelt es sich meist um Rezidive oder den persistierenden *Unguis incarnatus* mit einer Symptomdauer von mehr als 3 Monaten. Entsprechend den Stadien III und IV [19, 20] können hier zusätzlich chronische Deformitäten der Nagelplatte vorliegen. Bei diesen Patienten sehen wir die Durchführung einer Bildgebung als indiziert, um lokale knöcherne Veränderungen, die auf die Nagelmatrix drücken können, zu identifizieren [14].

## Therapie

Die Therapie sollte sich an den oben genannten Stadien orientieren. Die therapeutischen Strategien lassen sich in konservative Maßnahmen, nagelerhaltende Eingriffe und erweiterte chirurgische Maßnahmen untergliedern. Die Therapie hat das Ziel, ein Voranschreiten bzw. eine Chronifizierung zu vermeiden.

### Konservative Therapie

**Figure 3:**
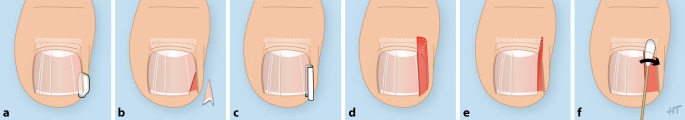

Bei Patienten mit akutem *Unguis incarnatus* und milder Ausprägung, d. h. ohne Granulationsgewebe, floride Inflammation oder relevanten lokalen Druckschmerz, empfehlen wir die Initiierung der konservativen Therapie (Abb. 2). Diese beinhaltet die lokale Druckentlastung (Adaptation des Schuhwerks, Zehenzwischenpolsterung) und Nagelpflege, inklusive der Behandlung einer ggf. vorhandenen Onychomykose [18, 21–23]. Durch Fußbäder, bis zu 3‑mal tägl., lässt sich nach Einweichen für 10–20 min der Nagelwall von der lateralen Nagelplatte mobilisieren. Anschließend können lokal topische Steroide oder desinfizierende Salben über einen Zeitraum von 1 bis 2 Wochen appliziert werden [18]. Zusätzlich kann die laterale Nagelplatte mittels Watte, Gaze oder Kompresse angehoben und abgepolstert werden, um den Druck auf den Nagelwall zu reduzieren (Abb. 3a; [18, 21]). Des Weiteren werden redressierende Tape-Verbände empfohlen, durch die der betroffene Nagelwall mit Pflasterzügeln zur Seite gehalten wird [24]. Allerdings ist die Applikabilität bei aktiven und berufstätigen Patienten limitiert.

Diese Maßnahmen müssen konsequent für 2 bis 12 Wochen, je nach Ausprägung und Therapieansprechen, fortgeführt werden [11, 18]. Dafür empfiehlt sich die Anbindung des Patienten an eine Podologie, die ggf. weitere Maßnahmen, wie z. B. die Anpassung eine Zehenspange, einleiten kann [25].

### Operative Therapie

Bei Patienten mit einem akuten *Unguis incarnatus* in moderater/schwerer Ausprägung oder mit einem chronischem *Unguis incarnatus* sollte eine chirurgische Therapie diskutiert werden (Abb. 2). Die chirurgischen Therapieansätze lassen sich in nagelerhaltende Eingriffe und erweiterte chirurgische Maßnahmen mit dauerhafter Verschmälerung der Nagelplatte untergliedern.

#### Nagelerhaltende Eingriffe (Notaufnahme)

Bei Patienten mit akutem *Unguis incarnatus* in moderater/schwerer Ausprägung, d. h. mit Schmerzen, florider Inflammation und/oder hypertrophem Granulationsgewebe, empfehlen wir die lokale Inspektion und Therapie in Leitungsanästhesie nach Oberst (LA) mit Blutsperre. Dieser Eingriff wird im aseptischen Bereich der Notaufnahme unter sterilen Kautelen durchgeführt. Im klinischen Alltag hat sich die Verwendung eines Handschuhfingers als Blutsperre etabliert [26]. Von einem Klemmen des Gummizügels wird abgeraten, da durch den hohen Druck Nervenschäden beschrieben sind [27]. Zudem sollte die Größe des Handschuhfingers dem Zehenumfang angepasst werden.

Zuerst sollte das hypertrophe Granulationsgewebe mit einem scharfen Löffel oder dem Skalpell entfernt werden [28]. Anschließend erfolgt die weitere Exploration des Raums zwischen Nagelplatte und Nagelwall. Sollte sich ein abszedierender Verhalt zeigen, kann dieser über denselben Zugang entlastet werden. Parallele Inzisionen am Nagelwall von lateral sollten nicht durchgeführt werden, da die verbleibende, häufig schmale, Hautbrücke nekrotisch werden kann. Zeigen sich in der Exploration Nagelreste oder Fremdkörper, müssen diese entfernt werden. Besonders ist auf ein spitzes laterales Nagelspikulum zu achten. In der Literatur werden in diesem Fall zwei verschiedene Vorgehen beschrieben [1, 11, 18]. Einige Autoren empfehlen die alleinige Glättung des Nagelspikulums, durch Entfernung eines Nagelkeils (Abb. 3b). Andere Arbeiten empfehlen die Schienung des lateralen Nagelspikulums mit einem Nagelröllchen (Abb. 3c; [29]). Dafür kann der Schutzschlauch der Nadel einer Butterfly-Flügelkanüle verwendet werden. Dieser sollte proximal abgeschrägt und längs gespalten werden. Anschließend wird die Schiene über das Spikulum und den gesamten Nagelrand geschoben und mittels Naht oder Steri-Strip auf dem Nagel fixiert. So kann die Schiene mit dem Nagel auswachsen [30, 31], der Nagelwall wird geschützt, und die Entzündung klingt ab [21, 31].

Wichtig ist, dass das Spikulum in diesem Fall nicht geglättet wird, da es der Schienung zusätzliche Stabilität verleiht [21, 31].

Für die alleinige Entfernung des Granulationsgewebes und die anschließende Glättung des lateralen Nagelspikulums sind Rezidivraten bis zu 30–70 % beschrieben [1, 32]. Da hier die sorgfältige Entfernung des lateralen Matrixhorn und der Nagelmatrix ausbleibt, kann die Nagelplatte weiter entlang des Nagelwalls wachsen und zur ständigen Irritation führen [33]. Für die Schienung mittels Nagelröllchen sind deutlich geringere Rezidivraten bis zu 10 % beschrieben [30, 34, 35]. Jedoch ist der Erfolg stark von der Compliance der Patienten abhängig.

#### Erweiterte operative Maßnahmen (Operationssaal)

Bei Patienten mit chronischem *Unguis incarnatus* ist nur bei florider Infektion eine sofortige Exploration indiziert (Abb. 2). Liegt diese nicht vor, sollte das weitere Vorgehen in enger Kooperation mit der Fußchirurgie abgestimmt werden. Hier bedarf es häufig erweiterter operativer Maßnahmen, die durch dauerhafte Verschmälerung der Nagelplatte das Einwachsen des Nagels verhindern. Diese Eingriffe sollten durch einen Facharzt im Rahmen einer elektiven ambulanten Operation durchgeführt werden.

In Deutschland kommt immer noch führend die Nagelkeilresektion (z. B. nach Emmert) zur Anwendung (Abb. 3d; [36]). Auch diese wird, ebenso wie die oben beschriebenen nagelerhaltenden Eingriffe, in LA und lokaler Blutsperre durchgeführt. Bei der Nagelkeilresektion erfolgen das Ausschneiden des erkrankten randständigen Zehennagelviertels sowie die Keilexzision von Nagelwall und Nagelbett mit vollständiger Entfernung der Nagelmatrix bis auf die Endphalanx der Zehe [36]. Allerdings sind Rezidivraten bis zu 20 % sowie schlechte ästhetische und funktionelle Ergebnisse beschrieben [37–39]. Ursächlich für die hohe Rezidivrate sind a.e. die unvollständige Resektion des lateralen Matrixhorns und der lateralen Nagelmatrix durch die keilförmige Exzision. Dies führt zu bizarren und schmerzhaften Nagelspikula. Des Weiteren kann es zu Nagelplattendeviation in Narbenrichtung kommen. Weiterhin problematisch sind die oft ausgeprägten postoperativen Schmerzen sowie mögliche Wundheilungsstörungen. Hier sind Infektionsraten bis zu 20 % beschrieben [5, 21, 40]. Aus diesen Gründen wird diese Operationstechnik kritisch diskutiert [21, 37–39, 41–43].

Heutzutage wird von vielen Autoren die schonendere selektive Nagelmatrixablation als Behandlungsstandard angesehen [11, 17, 18, 21, 39, 40, 44–46]. Das Ziel ist die selektive Entfernung des lateralen Nagelrands, inklusive des Matrixhorns und der dazugehörigen Nagelmatrix. Im Gegensatz zur Emmert-Plastik erfolgt keine Entfernung des Nagelwalls. Für die selektive Nagelmatrixablation sind zwei verschiedene Techniken beschrieben: die chirurgische oder chemische Ablation der Nagelmatrix.

Bei der chirurgischen Ablation erfolgt, analog zur Emmert-Plastik, das Setzen eines Hautschnitts in Verlängerung des Nagelwalls nach proximal bis kurz vor das (distale) Interphalangealgelenk (Abb. 3e). Alternativ kann die Inzision nach lateral geschwungen durchgeführt werden [21]. Anschließend wird der Nagel vom lateralen Nagelwall und vom Nagelbett gelöst. Dann erfolgt die Identifikation der Grenze zwischen gesundem und erkranktem Nagel. An dieser Grenze wird an der distalen Nagelplatte das Skalpell (15er-Klinge) mit nach oben gerichteter Klinge angesetzt. Dann wird das Skalpell, geführt durch die Nagelfaserrichtung, über die zuvor durchgeführte Hautinzision, bis an das proximale Ende der Nagelmatrix gedrückt. Dadurch wird zum einen das Nagelbett geschont, zum anderen wird der zu resezierende laterale Nagelteil, inklusive Nagelhorn, klar definiert. Dieser Nagelstreifen wird in toto entfernt. Schließlich erfolgt das sorgsame Entfernen des dazugehörigen Nagelmatrixepithels mit dem scharfen Löffel. Der proximale Hautschnitt wird mit 2 Einzelknopfnähten verschlossen.

Bei der chemischen Ablation der Nagelmatrix kann auf den proximalen Hautschnitt vollständig verzichtet werden (Abb. 3f). Nach Mobilisation des lateralen Nagelwalls wird nun das proximale Nagelhorn luxiert. Anschließend wird die Grenze zwischen gesundem und erkranktem Nagel definiert und, analog zu der chirurgischen Exzision, der erkrankte Nagelstreifen reseziert. Das Nagelmatrixepithel wird nun nicht chirurgisch, sondern chemisch abladiert. Dafür wird zuerst der proximale Nagelwall stumpf mobilisiert und anschließend das Nagelmatrixepithel mit in Phenol getränkten sterilen Wattestäbchen abladiert. Das Nagelmatrixepithel wird unter drehenden Bewegungen für je 3‑mal 1 min unter Druck mit dem Wattestäbchen eingerieben [5, 47]. Dabei sollten die Drehbewegungen von der Nagelplatte weg, hin zum ipsilateralen Nagelwall erfolgen, um eine Schädigung der medialen Nagelmatrix sowie des Restnagels zu verhindern. Die Phenolisierung verursacht eine kontrollierte Nekrose des Matrixepithels und des darunter liegenden Bindegewebes [21]. Der Situs muss zum Zeitpunkt der Phenolapplikation bluttrocken sein, da das Phenol sonst lediglich die Blutproteine koaguliert und seine Wirkung nicht am Matrixepithel entfaltet. Abschließend folgt die Spülung des Situs mit NaCl‑, Ringer-Lösung oder medizinischem Alkohol zur Verdünnung des Phenols am Auftragungsort [48].

In der Literatur wird die Überlegenheit einer der beiden Methoden kontrovers diskutiert. Mehrere Studien konnten zeigen, dass die chirurgische Resektion zwar zu weniger Rezidiven führt, die chemische Ablation aber insgesamt bessere postoperative Ergebnisse erzielt [44, 49]. Die Rezidivraten werden meistens zwischen 5 und 18 % angegeben [18, 44, 45, 49–52]. Ein Cochrane Review aus 2012 von Eekhof et al. (24 Studien, 2826 Patienten) kam, allerdings bei eingeschränkter Studienlage, zu dem Schluss, dass die chemische Ablation der chirurgischen Resektion hinsichtlich der Rezidivrate überlegen ist [17]. Allerdings kritisieren einige Autoren, dass die chemische Ablation in stark sezernierenden Wunden resultieren kann. Dies ist wiederrum mit einer erhöhten Infektionsrate assoziiert [40, 53]. Zudem wird kontrovers diskutiert, ob die Phenolapplikation ein gesundheitliches Risiko für den Anwender darstellt [54–56]. Aus diesen Gründen empfehlen wir, auch aufgrund der Tatsache der ausgewiesenen chirurgischen Expertise an unserem Haus, die selektive chirurgische Nagelmatrixresektion.

Zahlreiche weitere Verfahren sind beschrieben, werden in der Routine jedoch nicht eingesetzt. Beispielsweise erfolgt der Einsatz von CO_2_-Lasern und Elektrokautern zur Nagelmatrixentfernung [11, 18, 57–59]. Auch kann der laterale Nagelwall elliptisch oder halbmondförmig exzidiert und readaptiert werden. Dadurch wird eine plane Fläche und damit Raum für die laterale Nagelplatte geschaffen [60, 61]. Totale Nagelextraktionen sind beschrieben, werden heutzutage aber nicht mehr empfohlen [21].

### Additive Antibiotika

Die Notwendigkeit einer additiven Antibiose wird regelmäßig diskutiert. Allerdings konnten mehrere Studien zeigen, dass weder die orale noch die topische Antibiotikatherapie einen Einfluss auf das Behandlungsergebnis haben [4, 11, 17]. Wir verwenden keinerlei topische Antibiose. Die orale Antibiose sehen wir lediglich bei ausgewählten Fällen mit putridem, abszedierenden Geschehen als indiziert an. In diesen Fällen sollten Antibiotika mit einer guten Gewebegängigkeit, z. B. Clindamycin, verwendet werden [62].

### Aufklärung

Die Dokumentation und Aufklärung haben einen zentralen Stellenwert in der Therapie. Bei der konservativen Therapie sollten die Maßnahmen und Dauer ausführlich dokumentiert werden. Sowohl bei den nagelerhaltenden Eingriffen als auch bei den erweiterten operativen Maßnahmen ist eine Aufklärung zwingend notwendig. Sofern es der medizinische Befund zulässt, müssen die geforderten medikolegalen Zeitfristen der chirurgischen Aufklärung, die mindestens 24 Stunden vor dem Eingriff erfolgen soll, eingehalten werden. Die Aufklärung sollte, unabhängig vom Operationsverfahren, folgende Punkte beinhalten: Blutung und Nachblutung, Infektion, Wundheilungsstörung, Nagelbildungs- und Nagelwachstumsstörung, dauerhafte Verschmälerung der Nagelplatte, das Verbleiben von Nagelmatrixgewebe und die Bildung von schmerzhaften Nagelspornen, Rezidive, Folgeeingriffe, Narbenbildung und ein resultierendes unästhetisches Erscheinungsbild. Über mögliche Nervenverletzungen (traumatisch, toxisch) sowie allergische Reaktionen durch die Leitungsanästhesie sollte ebenfalls aufgeklärt werden.

## Schlussfolgerung

Der *Unguis incarnatus *ist ein häufiges Krankheitsbild. Die Therapie umfasst konservative sowie operative Maßnahmen. Diese sollten stadiengerecht angewandt werden. Essenziell für die erfolgreiche Behandlung des *Unguis incarnatus* ist die korrekte Indikationsstellung. Im Rahmen dieser Arbeit wird ein entsprechender praktischer Diagnose- und Behandlungsalgorithmus postuliert (Abb. 2). Die konservative Therapie sollte bei akutem *Unguis incarnatus* mit milder Ausprägung konsequent durchgeführt werden und zeigt gute Behandlungsergebnisse. Nagelerhaltende Eingriffe kommen bei moderaten/schweren Formen zum Einsatz. Der chronische *Unguis incarnatus* ohne floride Infektion stellt eine elektive Operationsindikation dar. In den meisten Fällen führen wir die selektive chirurgische Nagelmatrixresektion durch. Bei allen chirurgischen Maßnahmen muss die konsequente chirurgische Aufklärung erfolgen.

## Fazit für die Praxis

- Der eingewachsene Zehennagel ist keine Banalität.
- Bei akutem *Unguis incarnatus* mit milder Ausprägung sollte die konservative Therapie erfolgen.
- Bei akutem *Unguis incarnatus* mit moderater/schwerer Ausprägung werden nagelerhaltende Eingriffe in Lokalanästhesie empfohlen.
- Chronische Fälle ohne floride Infektion erfordern erweiterte chirurgische Maßnahmen.
- Die Emmert-Plastik wurde in den meisten Fällen durch die chirurgische oder chemische Nagelmatrixablation ersetzt.
- Jeder chirurgische Eingriff bedarf einer suffizienten chirurgischen Aufklärung.
